# Visceral Larva Migrans in Immigrants from Latin America

**DOI:** 10.3201/eid1707.101204

**Published:** 2011-07

**Authors:** Maria-Carmen Turrientes, Ana Pérez de Ayala, Francesca Norman, Miriam Navarro, José-Antonio Pérez-Molina, Mercedes Rodriquez-Ferrer, Teresa Gárate, Rogelio López-Vélez

**Affiliations:** Author affiliations: Ramón y Cajal Hospital, Madrid, Spain (M.-C. Turrientes, A. Pérez de Ayala, F. Norman, M. Navarro, J.-A. Pérez-Molina, R. López-Vélez);; Institute de Salud Carlos III, Madrid (T. Gárate, M. Rodriquez-Ferrer)

**Keywords:** toxocariasis, visceral larva migrans, immigrants, parasites, zoonoses, Latin America, dispatch

## Abstract

To determine whether increased migration is associated with an increase in incidence of toxocariasis (visceral larva migrans), we analyzed clinical data obtained from immigrants from Latin America. Although infection with *Toxocara* sp. roundworm larvae is distributed worldwide, seroprevalence is highest in tropical and subtropical areas.

Human toxocariasis is a zoonosis caused by the larvae of *Toxocara* sp. roundworms. Although distribution is worldwide, seroprevalence is highest in tropical countries, including in Latin America. Immigration from tropical areas to Europe continues to increase, with Spain a frequent destination. Data on visceral larva migrans (VLM) among immigrants from Latin America in western countries (primarily European countries, the United States, and Canada) are scarce. To determine whether increased migration is associated with increased VLM incidence, we analyzed clinical and epidemiologic data from immigrants from Latin America.

## The Study

We analyzed information about 634 immigrants from Latin America seen at the Tropical Medicine Unit of the Ramón y Cajal Hospital in Madrid, Spain, during April 1989–June 2008. Immigrants who were visiting friends and relatives were excluded. Patients with VLM were identified.

We used 5 strict criteria for diagnosing VLM: 1) positive serologic test for *Toxocara* sp. roundworm infection, performed by using a commercial ELISA *Toxocara* immunoglobulin (Ig) G Ridascreen (R-Biopharm GmbH, Darmstadt, Germany), following the manufacturer’s recommendations; 2) absolute peripheral blood eosinophil count >500 cells/mm^3^; 3) exclusion of other parasites causing eosinophilia, such as intestinal nematodes, particularly *Strongyloides stercoralis* (excluded by larval culture and serology by ELISA IgG), *Schistosoma* sp*.*, *Fasciola hepatica*, *Trichinella spiralis*, *Taenia solium*, *Echinococcus granulosus*, and cutaneous and blood microfilariae; 4) symptoms associated with VLM (respiratory signs, such as asthma, dyspnea, and eosinophilic pneumonia; dermatologic symptoms, including pruritus and recurrent urticaria; and abdominal symptoms, including abdominal pain and hepatomegaly); and 5) response to treatment with albendazole (10–15 mg/kg/d in 2 doses orally for 5 days) assessed 6 months after treatment, decreased titers to *Toxocara* sp. roundworm infection, decreased eosinophil count, and clinical improvement or resolution of symptoms.

The most frequent countries of origin for patients were Ecuador 221/634 (34.9%), Bolivia 176/634 (27.8%), Peru 71/634 (11.2%), and Colombia 56/634 (8.8%). Median age was 32 years (range 4–40 years); 421 (66.4%) patients were male. The median number of months from arrival in Spain to first consultation at the Tropical Medicine Unit was 19 months.

Eosinophilia was present in 135 (21.3%) patients. *Toxocara* antibodies were detected by ELISA in 31 (4.9%) patients. Concomitant serologic results positive for *Toxocara* sp. roundworm infection and eosinophilia were found in 28 (4.4%) patients; 606 patients were excluded. Of these 28 patients, 11 were excluded because of other concomitant parasitic infections that also can cause eosinophilia: 8 patients had positive ELISA results for *S. stercoralis* nematodes (not detected in fecal samples or larval culture); 1 had *Ascaris lumbricoides* eggs in feces; 1 had a positive indirect hemagglutination result but negative ELISA result for *E. granulosus* tapeworm; and 1 had a positive ELISA serologic result for *T. spiralis* nematodes. Another 12 patients were not included because detection of *Strongyloides* antibodies was not attempted. Only 4 of the 5 remaining cases fulfilled the strict inclusion criteria (Table); 1 patient was asymptomatic. After 6 months of treatment with albendazole, titers for *Toxocara* sp. roundworm infection and eosinophil count decreased, and symptoms improved or resolved for the 4 patients. Symptoms developed 3–18 months after arrival in Spain.

**Table:** Descriptions of 4 cases of visceral larva migrans in immigrants from Latin America, Spain, April 1989–June 2008*

Case no.	Age, y/sex	Origin	Clinical signs and symptoms	Chest radiograph results	Eosinophil count, absolute/mm^3^ (%)	6-mo follow-up		
						Eosinophil count/mm^3^	Decrease in antibody titers	Symptoms
1	28/M	Bolivia	Asthma-like syndrome†	Slight right parahiliar infiltrate	700 (17.0)	500	Yes	None
2	29/F	Dominican Republic	Dry cough, dyspnea, chest pain, eosinophilic pneumonia	Bilateral alveolar infiltrates	1,400 (10.5)	600	Yes	None
3	5/F	Ecuador	Asthma-like syndrome, abdominal pain	No findings	1,050 (15.0)	700	Yes	None
4	40/F	Colombia	Abdominal pain	Not done	1,500 (14.8)	400	Yes	Clinical improvement

*All patients were treated with albendazole (10–15 mg/kg/d in 2 doses orally for 5 days). †Wheezing and dry cough.

Clinical toxocariasis is rarely diagnosed in western countries as previously described despite evidence of environmental exposure (*1*). Results of seroprevalence surveys performed in healthy adults in France were positive for 2%–5% of persons in urban areas, compared with 14%–37% in rural areas (*2*). In Latin America, rates vary from 1.8% to 51.6% (*3**,**4*). However, literature references to VLM imported by immigrants are scarce (*5*), and the disease may be underdiagnosed in the immigrant population, partly because of nonspecific symptoms and the limitations of serologic diagnosis. In our study, serologic prevalence of *Toxocara* antibodies was 4.9% (31/634).

Toxocariasis is a common cause of eosinophilia in peripheral blood, although its absence does not exclude infection by *Toxocara* sp. roundworms. In other studies, 27% of patients had reactive serologic results for *Toxocara* sp. roundworm infection without eosinophilia (*6*); similarly, 27% of patients with high antibody titers had eosinophil counts within the reference range (*7*). By including only patients with eosinophilia, our study applied more stringent criteria. Thus, 28 (90%) of 31 patients who had positive serologic results showed an elevated eosinophil count, in accordance with previously described high *Toxocara* sp. roundworm seroprevalence (<68%) in patients with eosinophilia of unknown cause (*8*).

Eleven of the 28 patients with positive serologic results for *Toxocara* sp. roundworm and eosinophilia also had positive serologic results for other parasites that cause eosinophilia. One patient who was infected with *A. lumbricoides* roundworm had asthma, hepatomegaly, and pruritus. The latter is not usually associated with this parasite, which suggests possible co-infection.

Serologic tests for *Toxocara* sp. roundworm infection should be interpreted with caution because commercial ELISA kits that use excretory and secretory antigens derived from second-stage larvae of *Toxocara* sp. roundworms exhibit a sensitivity of 91% and a specificity of 86%; cross-reactivity has also been described with other nematode infections. The positive serologic results for *T. spiralis* nematodes and *E. granulosus* tapeworms may have been caused by cross-reactivity (*9*). These patients had asthenia and asthma, respectively, and symptoms resolved after treatment with albendazole. Eight patients with *Strongyloides* antibodies were also excluded; however, this finding does not exclude co-infection by both parasites. Finally, a limitation of the study was that we could not definitively exclude cryptic strongyloidiasis for 12 patients because of the difficulty in finding *S. stercoralis* threadworms in feces and because detection of *Strongyloides* antibodies was not possible.

Other authors have already recommended caution when interpreting positive *Toxocara* sp. roundworm serologic results in asymptomatic persons or persons with equivocal symptoms (*10*). Therefore, the asymptomatic patient with positive serologic and eosinophilia results was also excluded.

This study illustrates the difficulties in diagnosing VLM in immigrants from tropical and subtropical areas of Latin America because only a very small proportion of patients in the series (n = 4) had VLM. The most common symptoms were respiratory (3/4); 2 patients had asthma-like syndrome and 1 had chest pain followed by abdominal pain (2/4). Typical manifestations of VLM are abdominal symptoms (pain, hepatomegaly) and respiratory symptoms (severe asthma, eosinophilic infiltrates). In addition to this, evidence points to *Toxocara* sp. roundworm infection as a risk factor for asthma in some populations (*11**,**12*).

Albendazole is the treatment of choice for VLM; for practical purposes, it could be recommended for presumptive treatment in immigrants from Latin America with eosinophilia in whom strongyloidiasis is suspected (*13*). However, the superiority of ivermectin over albendazole has been documented in the treatment of chronic strongyloidiasis (*14*).

## Conclusions

VLM may be difficult to diagnose, especially in immigrants from regions in Latin America where polyparasitism is endemic. Positive serologic test results, marked eosinophilia, absence of other helminthic infections, compatible clinical signs, and disappearance of symptoms after specific treatment can help establish a VLM diagnosis, especially in areas of low parasitism. VLM should be included in the differential diagnosis of eosinophilia in immigrants (children and adults) from tropical areas if respiratory or abdominal symptoms are evident. Albendazole is an effective and relatively safe drug that could be used to treat suspected VLM and other concomitant nematode infections, including cryptic *S. stercoralis* threadworm infections. Empirically described treatment may lead to resolution of clinical symptoms, even though ivermectin is a better treatment for chronic strongyloidiasis.
